# The Evidence Surrounding Non-Alcoholic Fatty Liver Disease in Individuals with Cancer: A Systematic Literature Review

**DOI:** 10.3390/curroncol30010005

**Published:** 2022-12-21

**Authors:** Elena S. George, Surbhi Sood, Nicole Kiss, Robin M. Daly, Amanda J. Nicoll, Stuart K. Roberts, Brenton J. Baguley

**Affiliations:** 1 Institute for Physical Activity and Nutrition, School of Exercise and Nutrition Sciences, Deakin University, Geelong, VIC 3125, Australia; 2 Gastroenterology Department, Alfred Health, Prahran, VIC 3004, Australia; 3 Allied Health Research, Peter MacCallum Cancer Centre, Melbourne, VIC 3000, Australia; 4 Gastroenterology Department, Eastern Health, Box Hill, VIC 3128, Australia; 5 Central Clinical School, Monash University, Clayton, VIC 3800, Australia

**Keywords:** non-alcoholic fatty liver disease, non-alcoholic steatohepatitis, metabolic syndrome, cancer treatment, chemotherapy

## Abstract

Emerging evidence indicates an association between non-alcoholic fatty liver disease (NAFLD), cancer development and mortality. Cancer treatment-induced metabolic and hepatic dysfunction may be associated with increased rates of NAFLD. The review aims to investigate current evidence surrounding NAFLD in adults (≥18 years) with cancer including prevalence, effect of cancer treatments, metabolic co-morbidities, and mortality. Embase, Scopus, PubMed, and CINAHL were searched from inception to December 2021 including randomized controlled trials and observational studies. Twenty-three articles were included, comprising 142,218 participants. The overall risk of bias for observational studies was determined as low for 10 studies and neutral for 12 studies, and the RCT was determined as some concerns. The prevalence of NAFLD, based on imaging or histology, in adults with cancer ranged from 0.5 to 81.3%, with higher prevalence in breast, colorectal and gynecological cancers. Higher rates of NAFLD were also seen in patients who (i) underwent treatments—including chemotherapy and hormone therapy and/or who (ii) had higher BMI or other metabolic co-morbidities. NAFLD was associated with an increase in all-cause and cancer-related mortality. Based on review results, it is recommended that further assessment is carried out to determine whether liver screening in high-risk patients is cost effective and if interventions can be implemented to improve hepatic and health outcomes in adults with cancer.

## 1. Introduction

Non-alcoholic fatty liver disease (NAFLD) is the most common cause of chronic liver disease worldwide, affecting 5.5 million Australians, and 1 billion people globally [1,2]. The disease exists on a spectrum ranging from simple steatosis, non-alcoholic steatohepatitis (NASH), and with increased inflammation and fibrosis can lead to cirrhosis and elevated risk of liver cancer and cardiovascular diseases [3]. However, rates may be underestimated as the prevalence and incidence of recorded NAFLD diagnosis in healthcare records are lacking [4]. This may be as a result of underreported new and existing cases, differing definitions and diagnosis methods, and limited studies undertaken to elucidate rates [2]. NAFLD occurrence is reported to be highest in populations with metabolic syndrome (MetS) and existing chronic diseases affecting up to 80% of people with type 2 diabetes (T2DM) and up to 95% of obese people [2,5]. NAFLD co-exists with components of the MetS, such that it is often referred to as the hepatic manifestation of the metabolic syndrome [2]. Ninety percent of patients with NAFLD have more than one feature of metabolic syndrome, reflecting the high prevalence [6,7].

Cancer is the second leading cause of death globally, with the burden estimated to have risen to 18.1 million per annum, including 9.6 million deaths in 2018. [8] Chronic diseases such as the MetS, its components and thus likely NAFLD, may increase the risk of cancer [9]. Relative five-year survival rates are highest for prostate (~96%) and female breast cancer (~91%) [10,11] in Australia, yet androgen-deprivation therapy (ADT) for prostate cancer and endocrine therapy for breast cancer along with chemotherapy are all associated with increased metabolic dysfunction and heightened rates of cardiovascular disease (CVD) [12]. Therefore, all of these treatments may have negative effects on the liver. This is attributable to both the increased risk of cancer due to pre-existing metabolic and hepatic dysfunction as reported previously [13], but also potential long-term cardiometabolic side effects from cancer treatments which likely add an additional risk to adverse metabolic outcomes and NAFLD [14]. Radiotherapy, chemotherapy and hormonal therapy (e.g., tamoxifen) have all been shown to induce MetS while the effects on the liver have not been well established [15]. Furthermore, treatment-induced metabolic dysfunction leading to MetS is well documented in adults with cancer; however, the rates of NAFLD are unknown and largely overlooked [16]. Given that some hormonal, chemotherapy, and radiotherapy cancer treatments can be hepatotoxic, this is likely an important clinical population who would benefit from screening for liver injury. 

The aim of this systematic review is to determine the (i) prevalence of NAFLD in adults with cancer, (ii) to determine whether there is development or worsening of NAFLD with treatment and (iii) any impact on cancer-related mortality. Furthermore, this review aims to identify metabolic parameters that may predispose to NAFLD to help characterize high risk sub-groups.

## 2. Materials and Methods

All methodology was specified prior to the literature search and documented in a protocol registered with PROSPERO (CRD42021242186). The review was conducted according to Preferred Reporting Items for Systematic Review and Meta-Analysis (PRISMA) guidelines 2020 (Table S1) [17].

### 2.1. Search Strategy

Electronic search was conducted to identify peer-review articles published up to and including 17 December 2021. The four databases searched were Embase, Scopus, PubMed and CINAHL. The exact search strategy was tailored to each database but included a combination of relevant search terms relating to (a) adults with a diagnosis of cancer, and (b) liver outcomes of NAFLD and/or NASH. The complete search strategy for each database is provided in Appendix A. Individual cancer terms were included based on the most common cancer types reported by the National Cancer Institute [18,19]. The list of references, relevant original studies or reviews were also hand searched for relevant papers. Title and abstract screening were completed in duplicate by three independent authors (ESG, SS and BJB) using Covidence systematic review software (Veritas Health Innovation, Melbourne, Australia). Full-text screening was carried out by the same authors, and any conflicts were resolved through consensus of at least two authors (ESG, SS, BB). The research question was defined according to the PICOS (Participants, Intervention, Comparison, Outcomes and Study Design) scheme presented in Table 1.

### 2.2. Eligibility Criteria 

The inclusion criteria for this systematic review followed the PICOS framework [17]. Studies were considered eligible for inclusion if (a) the article was published in English; (b) participants were men or women aged 18 years and older; (c) participants had a diagnosis of cancer; and (d) the study reported liver outcomes for NAFLD and/or NASH (based on ultrasonography, Magnetic resonance imaging (MRI), abdominal Computed tomography scan (CT), ICD-9 codes or histology). We included the following study designs: randomized and non-randomized clinical trials, cohort, cross-sectional or case-control study design. Case studies were excluded. 

### 2.3. Data Extraction and Quality Assessment and Risk of Bias

Data extraction was completed by one author (SS) and verified by two independent authors (ESG and BJB). Information extracted from each study included study design and duration of the study, participant characteristics (number of participants (n), age, BMI, sex), cancer type and treatment, NAFLD and/or NASH diagnosis and classification of liver disease, number of NAFLD participants (n, %), primary study outcomes and main findings. The quality assessment of included studies was conducted in duplicate by three independent authors (ESG, SS and BJB). Data were extracted from referenced protocols to inform the quality of the study when completing the risk of bias assessment. The Academy of Nutrition and Dietetics Evidence Analysis Library Quality Criteria Checklist was utilized for observational studies [20]. This checklist consists of an evaluation of studies’ relevance (four questions) and validity (ten questions). Based on these criteria, the researchers assigned each article a quality rating of positive, neutral or negative (+, Ø, −) depending on the rating (Yes, No, Unclear, N/A) given to each individual question. Conflicts on ratings were resolved through consensus between at least two authors. Studies scoring a rating of positive were considered to be high-quality studies. The quality of randomized controlled trials was assessed using the Revised Cochrane Risk of Bias tool (RoB 2.0) [21]. This tool consists of five domains assessing the risk of bias arising from the randomization process, risk of bias due to deviations from the intended protocol, missing outcome data, measurement of the outcome, and selective reporting [21]. The researchers answered signaling questions to assign a domain-level judgement about the risk of bias (low risk of bias, some concerns, and high risk of bias). 

## 3. Results

**Figure 1:**
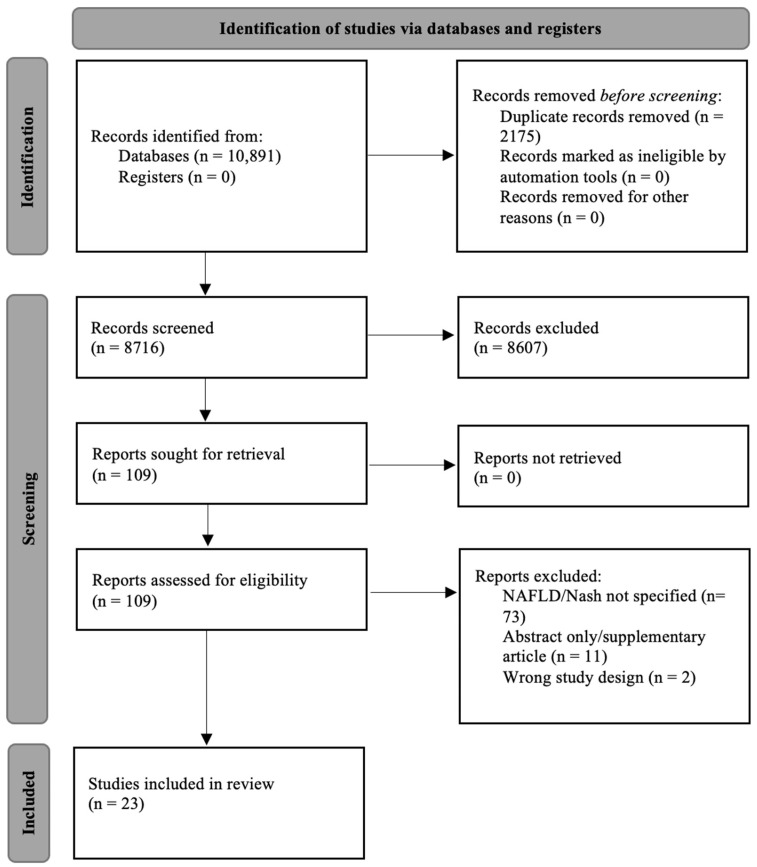
Preferred reporting items for systematic review and meta-analysis (PRISMA) statement flow diagram.

The literature search process is shown in Figure 1. The search identified a total of 10,891 articles from the databases. Duplicates (n = 2175) were removed, and the remaining 8716 records were identified for title and abstract screening. Among these, 8607 articles were deemed ineligible as a result of title or abstract screening. One hundred and nine studies from the search were eligible for full-text screening, and 86 were excluded for the following reasons: NAFLD or NASH not specified as an outcome (n = 73), abstract only/supplementary articles (n = 11) and wrong study design (n = 2). Therefore, 23 studies were included in this systematic review. 

### 3.1. Study Characteristics and Qualitative Assessment 

The characteristics of the 23 articles included are presented in Table 2. Eleven articles reported results from cohort studies [22,23,24,25,26,27,28,29,30,31,32] of which seven studies were retrospective [23,24,26,29,30,31,32], four articles were cross-sectional studies [33,34,35,36], seven from case control studies [37,38,39,40,41,42,43] and one article was a randomized controlled trial [44]. The articles were published between the years 2002 and 2020. Of these, five were conducted in the United States [22,24,30,36,41], four in Europe [27,28,34,44], four in Japan [25,40,42,43], two in Turkey [38,39], Taiwan [26,31] and Korea [32,33], one in each of the following countries Canada [23], Philippines [29], Iran [35] and Israel [37]. Sample size ranged from 19 [34] to 82,938 [28] participants and age ranged from 18 [24] to 76 years old [28]. The gold standard diagnostic modality for NAFLD and NASH is liver biopsy. However, noninvasive and inexpensive approaches such as imaging and biochemical outcomes are commonly used in research and clinical practice to measure patients with liver disease [45]. NAFLD was defined using a range of methods, with ultrasonography used to characterize hepatic steatosis in eight studies [22,25,26,31,35,38,42,44], CT scans in eleven studies [23,24,28,30,34,37,39,40,41,43], MRI in two studies [29,33], International Classification of Disease codes in one study ICD-9 and ICD-10 [36], Danish National Registry in one study, which is based on the ICD-10: K74.6 (other and unspecified cirrhosis of liver) [27], and the Hepatic Steatosis Index in one study [32]. While three studies reported on liver enzymes, this was done in addition to ultrasound and MRI [22,33,44]. The main study outcomes related to NAFLD, and treatment modalities, survival and mortality and metabolic co-morbidities are shown in Table 3.

The prevalence of NAFLD in all adults with cancer ranged from 0.5% (n = 259 out of 51,821) [28] in individuals in prostate cancer, to 81.3% (n = 52 out of 64) [44] in endometrial cancer. Breast, colorectal and hepatocellular cancer were the most frequently investigated cancer types reporting NAFLD prevalence in nine, four and three studies, respectively.

### 3.2. The Effect of Cancer Treatment on NAFLD

Fifteen out of 23 studies reported treatment methods that were associated with the development of NAFLD and/or NASH across multiple cancer types including breast, ovarian, gastric, HCC, infiltrative HCC, endometrial and colorectal including 138,138 participants (range: 56 [43] to 39,840 [27]) [22,26,27,28,29,30,31,32,35,36,38,40,41,42,43,44].

- Chemotherapy and NAFLD

Four studies assessed chemotherapy and NAFLD prevalence, including a total of 12,225 participants (range: n = 152 [35] to 11,187 [36]) in breast, gastric and hepatocellular types [35,36,38,40]. The studies included in this review reported on adjuvant chemotherapy with S-1 (80 mg/m^2^/day), systemic chemotherapy, trans arterial chemoembolization, and FOLFOX chemotherapy. Liver disease has been previously reported following treatment with chemotherapy such as methotrexate, and 5-FU [46,47]. One of these studies was conducted in breast cancer patients, reporting that patients treated with chemotherapy did not have a direct correlation with hepatic steatosis (r = 0.14, *p* = 0.17) [38]. Two studies in patients with gastric cancer receiving chemotherapy showed it was associated with a significantly increased risk of NAFLD as indicated by the frequency of fatty liver increasing from 2% to 46.7% in all patients after chemotherapy treatment (*p* = 0.0001) [35], and with adjuvant chemotherapy the risk of NAFLD and decompensated cirrhosis was increased (OR 28.26, 95% CI: 8.55–93.37; *p* < 0.001) [40]. Overall, three out of four studies concluded that chemotherapy treatment increases risk of NAFLD development.

ii Hormone therapy and NAFLD

Six studies evaluated hormone therapy and NAFLD including a total of 83,983 participants [26,28,31,32,43,44]. Studies conducted in breast and prostate cancer undergoing hormone therapy reported an increased risk of NAFLD, including aromatase inhibitors (HR: 15.92; 95% CI, 6.56–38.63; *p* = 0.0001) [32] and ADT (HR: 1.54, 95% CI, 1.40–1.68, *p* < 0.001) [28], respectively. Additionally, patients who underwent treatment with ADT had a higher prevalence of liver cirrhosis (HR 1.35, 95% CI 1.12–1.60, *p* = 0.015) and liver disease after treatment (HR: 1.84, 95% CI 1.73–1.96; *p* < 0.0001) [28]. Four out of six studies reported on patients undergoing cancer treatment with tamoxifen [26,31,43,44]. Two studies reported tamoxifen-related NAFLD in patients with a BMI of greater than or equal to 22 kg/m^2^ (HR, 1.58; 95% CI: 1.00–2.48; *p* < 0.05) [26] and significantly higher risk of newly developed fatty liver (HR: 3.69, 95% CI 1.67–8.13, *p* < 0.001) [31]. Another study in overweight and obese patients with endometrial cancer receiving tamoxifen [44], indicated NAFLD occurrence was higher in the treatment group compared to placebo group (log rank *p* = 0.017). Conversely, one study with patients undergoing tamoxifen treatment as adjuvant endocrine therapy reported no significant results in patients with NASH [43].

iii Surgery and NAFLD

Three studies, including a total of 12,776 participants with endometrial cancer and HCC, evaluated surgery and NAFLD [36,41,42]. Two studies reported on patients with endometrial cancer undergoing oophorectomy surgery with one study indicating significantly increased risk of NAFLD post-surgery (HR, 1.70; 95% CI, 1.01–2.86; *p* = 0.047) [42]. Another study, conducted in patients with endometrial cancer, reported NAFLD diagnosis increased to 25.4% after surgical management (HR, 0.29 95% CI (0.16–0.51, *p* < 0.001) [41].

### 3.3. Survival and Mortality

Six out of six studies (from overall 23 studies) including 52,073 participants, investigating mortality (range: 60 [23] to 39,840 [27]) determined that NAFLD increases the risk of all-cause mortality and cancer-related mortality in numerous cancers including HCC, breast cancer, endometrial, colorectal cancer, and a study including multiple types [22,23,27,29,32,36]. Two studies investigated patients with HCC indicating that the incidence of NAFLD significantly reduced survival time (9.43 months vs. 38.47 months) and increased mortality within two years (*p* < 0.001) [36]. Similarly, Prieto et al. reported that the survival time between those with and without liver cirrhosis was significantly different (9.4 months vs. 38.5 months, *p* ≤ 0.001) and survival differences were seen in treatment modalities (surgery, 13.17 months vs. transarterial chemoebolization and/or radiofrequency, 30.3 months vs. systemic chemotherapy, 26.7 months *p* ≤ 0.001) [29]. In two studies in patients with colorectal cancer, the first with 60 people reported that hepatocyte ballooning was linked with decreased hepatic disease-free survival (RR = 3.31, *p* = 0.003) [23] and the second study with 39,840 patients, with and without liver cirrhosis, showed that those with cirrhosis had a higher 30-day mortality at 24.1% in comparison to patients who were not cirrhotic (13.3%) and without liver disease (8.7%) (RR 2.59, 95% CI: 1.86–3.61) [27]. The remaining two studies assessed patients with breast cancer. Lee et al., including 253 patients undergoing aromatase inhibitor therapy which led to the development of NAFLD, reported a lower disease-free survival than those without NAFLD (HR, 2.8 95% CI: 1.26–6.23, *p* = 0.012) [32] and Brown et al. including 387 people with multiple cancer types reported an association with increased risk of all-cause (HR: 2.52, 95% CI: 1.47–4.34; *p* = 0.001) and cancer-specific mortality (and HR: 3.21, 95% CI: 1.46–7.07; *p* = 0.004) with NAFLD [22].

### 3.4. Metabolic Co-Morbidities

Five studies reported on BMI with a total of 2106 (range: 19 [34] to 875 [42]) participants investigating childhood-onset craniopharyngioma, gastric cancer, endometrial cancer, breast cancer and/or multiple cancer study (breast, gastrointestinal, genitourinary, gynaecological, lung and haematological) [22,33,34,40,42]. In four out of the five studies that reported on BMI as a risk factor for NAFLD in individuals with cancer, there was a positive association confirming the known association between increased BMI and NAFLD in the context of adults with cancer [22,33,34,40,42]. There were seven studies that assessed a range of metabolic co-morbidities in addition to NAFLD, including one or more of the following: type 2 diabetes mellitus, insulin resistance, higher fasting insulin levels, obesity, hypertension, hypercholesterolaemia, hypertriglyceridemia [22,24,27,33,34,38,42]. These included a total of 44,652 (range: 19 [34] to 39,840) participants [27] in studies including a combination of multiple cancer types, breast cancer, colorectal cancer, adults with a history of childhood onset craniopharyngioma and endometrial cancer. T2DM was a comorbidity reported in five studies in patients who developed NAFLD who had breast, colorectal and/or endometrial cancers [24,27,33,38,42]. Two out of five studies reported that T2DM was significantly associated with an increased risk of NAFLD in patients with breast cancer (OR = 11.87, 95% CI: 1.06–132.37; *p* = 0.004) [33] and endometrial cancer (HR, 1.41; 95% CI, 1.06–1.88) [42]. The remaining three studies suggested that patients with T2DM were more likely to have an increased risk of NAFLD, but no significance was reported [24,27,38]. Similarly, dyslipidaemia was a commonly reported factor in five studies and was associated with NAFLD in colorectal, endometrial, and breast cancer, and studies in multiple cancer types [22,27,33,38,42]. Two out of five studies confirmed that elevated triglycerides were significantly associated with NAFLD in multiple cancer types (2.6 vs. 1.6 mmol/L; *p* = 0.007) [22] and breast cancer (OR = 50.27; 95% CI: 4.41–573.03; *p* = 0.002) [33]. In addition, hypercholesterolaemia in endometrial cancer patients was significantly associated with NAFLD (HR: 1.90; 95% CI:1.26–2.87; *p* = 0.004) [42]. Two studies reported that no association was found between hypercholesterolaemia and steatosis [38,42]. One study investigated the link between blood pressure and NAFLD in participants across multiple cancer types, and this showed significantly higher systolic (130.1 vs. 121.8 mm Hg; *p* = 0.004) and diastolic (76.8 vs. 73.0 mm Hg; *p* = 0.029) blood pressure in patients with NAFLD [22]. Two studies demonstrated consistent evidence that obese individuals with breast [38] and colorectal cancer [24] were more likely to have an increased risk of NAFLD; however, no significance was reported.

### 3.5. Risk of Bias

The risk of bias assessment for all included studies is shown in Table 4 and Table 5. For the observational studies, 10 of the 23 articles received a positive quality rating, indicating a low risk of bias, and 12 articles received a neutral quality rating (Table 4). All observational studies were considered relevant and indicated applicability to practice. Validity in all 22 studies was also determined to be of high quality based on clear research questions, subject/patient selections, clearly defined research outcomes, use of valid and reliable measurements, and the reporting of limitations. For the single RCT study, we also evaluated the protocol referenced within the methods [48,49]. The risk of bias assessment was determined as some concerns. This was mainly due to missing information in one or more domains.

## 4. Discussion

This is the first systematic literature review, to our knowledge, to assess the evidence surrounding the prevalence of NAFLD in adults with cancer, the effect of therapy, and its impact on mortality. The main findings from this review were as follows: (1) the prevalence of NAFLD in adults with cancer varied widely but appeared highest in breast, gynaecologic and colorectal cancer; (2) a number of treatments were associated with an increased risk of NAFLD, including chemotherapy, tamoxifen for breast cancer, and hormone therapy in prostate cancer, and (3) NAFLD seems to poorly impact prognosis and increase mortality in people with cancer; finally, (4) individuals with higher BMI and other metabolic risk factors (irrespective of cancer diagnosis) appear to be at increased risk of NAFLD. Our review showed considerable heterogeneity in the methods of NAFLD diagnosis, sample size, study design, cancers and treatments and precludes definitive prevalence of NAFLD and associated risk factors in adults with cancer. Given the results from this review, targeted screening and/or assessment of NAFLD in those at higher risk appears to be warranted in individuals with cancer and should be assessed for cost effectiveness. It is recommended that future research prioritize supportive care survivorship interventions in adults with cancer.

Varying cancer treatments may be associated with the development of MetS phenotype and linked to pathophysiological factors that underpin the MetS. These dysfunctions include (i) insulin resistance and an increase in insulin-like growth factor 1, (ii) elevated adipokines secreted from visceral adipocytes, and (iii) free fatty acids and aromatase activity; these all collectively attribute to MetS and furthermore NAFLD [50]. These mechanisms as well as angiogenesis, glucose utilization, and oxidative stress with DNA damage, are thought to work together to increase the risk of NAFLD in adults with cancer [51]. Given the multiple metabolic alterations associated with NAFLD, high-risk patients that are diagnosed and treated for cancer and importantly present with multiple comorbidities may require liver screening, diagnosis and are likely to benefit from interventions for NAFLD.

MetS and obesity are associated with an increased risk of common cancers, albeit the risk seems to vary between populations and with the definition used for MetS [52]. Increased rates of MetS and CVD among patients diagnosed with cancer have been extensively reported in the literature [53]. Despite the large body of evidence assessing MetS in people with cancer [54], NAFLD and hepatic outcomes are not routinely assessed specifically. In this review, we have identified that there appears to be an increased risk of NAFLD in adults with cancer and this was amplified in those with a higher BMI. The use of chemotherapy in women with breast cancer and hormonal therapy for men with prostate cancer have been shown to contribute to an increase in body weight and fat mass, which are an established risk factor for NAFLD [55,56]. Multiple forms of chemotherapy have acute hepatocellular effects on liver dysfunction or toxicity, and previous studies have indicated that specific chemotherapy agents (i.e., 5-FU, platinum derivatives and taxanes) can lead to hepatic steatosis. Whilst steatosis composes NAFLD, the association of isolated chemotherapy agents or regiments (combination of agents) and risk of NAFLD is yet to be elucidated [57]. Pre-existing liver damage (from alcohol intake and/or poor lifestyle choices) prior to chemotherapy may have negative consequences in treatment tolerance [58]. Conversely, a reduction in lean (muscle) mass is often seen on presentation and over the duration of treatment in other cancer diagnoses including lung, upper and lower gastrointestinal cancers, prompting the National Cancer Institute to define Common Terminology Criteria for Adverse Events due to reduction in lean muscle mass (which mainly include altered liver enzymes) in all adults with cancer treated with chemotherapy [59]. Whilst adults with a higher BMI have an increased risk of hepatic steatosis, as reinforced by the studies in this review, we hypothesize that there may be a similar magnitude of liver damage in adults that experience reduced lean (muscle) mass, and are at risk of malnourishment as a consequence of chemotherapy. However, these cancer types have not been well investigated in the context of NAFLD and thus are not captured in this review. However, future studies may consider such assessments to determine whether the risk of NAFLD is also increased in these cancer types.

NAFLD has been described as a mediator for the obesity-cancer association [13], with the progression and onset of NAFLD underpinned primarily by insulin resistance. Subsequently, cancer treatment such as chemotherapy and hormone therapies are metabolized by the liver and therefore pose a risk of liver damage [59,60]. Therefore, whilst treatments are essential and efficacious, they appear to also elicit hepatotoxicity and the risk can be increased with pre-existing liver disease and extended treatment durations. Endocrine therapies in breast cancer and androgen depravation therapy in prostate cancer can last for many years (either continuously or intermittent). Even though these treatments are known to have a negative effect on the liver, this review has demonstrated that there are limited studies focused on NAFLD, and the cancer types investigated to date were heterogenous. Furthermore, the body of work assessing the impact of cancer treatment on metabolic risk factors and MetS is substantial [61,62], although these too overlook the hepatic implications and NAFLD. In addition to the direct adverse effects these therapies have on the liver, the treatments likely lead to an enhanced risk of NAFLD through an increase in body weight and/or reduction in lean mass, with lipid and glucose-insulin alterations [63]. Therefore, people with cancer and those with additional risk factors such as high BMI or reduced lean mass, who are at increased risk for NAFLD, should be considered for targeted screening and hepatic monitoring.

Damage to the liver increases the risk of liver-related and all-cause mortality [64]. Individuals with a history of cancer are a high-risk group for metabolic dysfunction and CVD, and the results from this review indicate an increased risk for NAFLD and its associated complications in cancer survivors. Therefore, it is important to further investigate this relationship and whether screening practices should be routinely carried out to monitor the liver in these individuals. Furthermore, rehabilitation involving lifestyle intervention, aimed at improving metabolic outcomes, should also consider hepatic benefits. This may prevent harmful side effects and damage to the liver in these individuals.

Future studies in individuals with cancer should consider assessment of liver outcomes and indeed long-term effects given the higher rates of MetS and increased mortality in these participants. Hormone therapy seen in breast and prostate cancer and some chemotherapy agents appear to be associated with increased liver steatosis and potentially NAFLD; however, future studies are warranted to evaluate the dose of treatment, pre-existing conditions, and liver outcomes. Furthermore, whether malnutrition, low lean muscle mass, and sarcopenia co-exist with NAFLD in individuals who have/had a cancer diagnosis requires additional investigation. Targeting high-risk adults with cancer and providing diet and exercise interventions which are simple, cost-effective ways proven to reduce metabolic health outcomes and mortality in other patient groups including those with heart disease, may be one approach [65]. Moreover, improving lifestyle is currently the only proven and safe way to improve hepatic outcomes [66]. Screening for liver disease and in particular NAFLD in high-risk cancer patients needs to be evaluated to determine the cost effectiveness (based on prevalence). However, to date, liver outcomes, the cost effectiveness of screening and the prevention and management of NAFLD in cancer patients have been largely overlooked.

The strengths of this systematic review are in its robust systematic methodology. Limitations include that the study designs were heterogenous, as were the cancer types captured. As a result, there was no scope for meta-analysis. Furthermore, NAFLD was assessed using a range of assessment and imaging techniques and no studies used the gold standard liver histology. However, such an invasive assessment for individuals already undergoing cancer treatment may not be appropriate. In addition, for those undergoing treatment, in many cases, the results could not be extrapolated due to the short follow-up time or lack of reporting. These are important outcomes to consider for future studies as the long-term effects of treatments in individuals with cancer are important to determine the effects of long-term liver and cardiovascular health.

## 5. Conclusions

People with cancer may have a higher risk of NAFLD and certain cancer treatments including chemotherapy, tamoxifen, and hormone therapies, seem to further exacerbate liver damage. High BMI and some metabolic risk factors appear to further increase the risk of NAFLD and those with NAFLD appear to have an increased risk of all-cause and cancer-related mortality. Further studies are needed to confirm if targeted hepatic screening in high-risk groups is cost effective and warranted to improve hepatic and health outcomes.

## Figures and Tables

**Table 1:** PICOS criteria for included studies.

Parameter	Criteria
Participants	Adults aged >18 years old, with a diagnosis of cancer.
Intervention	Treatment modalities, lifestyle factors and/or metabolic comorbidities.
Comparison	N/A
Outcomes	NAFLD and/or NASH measured by ultrasonography, abdominal computed tomography, liver magnetic resonance imaging, hepatic steatosis index, ICD-9 codes or histology
Study design	Randomized and non-randomized clinical trials, cohort, cross-sectional or case-control study design.

**Table 2:** Data extraction of the studies (n = 23) included in the systematic review.

Author, Year, Country	Study Design, Length of Study, Median Length of Follow-Up	Participant Characteristics (n, Age, BMI, Sex)	Cancer Type and Treatment	NAFLD Diagnosis and Classification of Liver Disease	n(NAFLD), %	Primary Study Outcomes	BMI Outcomes	Main Findings
Brown et al., 2017 United States [22]	Cohort study 17.9 years	n = 387 (cancer survivors) Age range: 20–74 years Mean age: 51.6 ± 1.11 BMI: 26.5 ± 0.4 F: 72%, M: 28%	Multiple cancer types Treatment: NR for each cancer type	Ultrasonography	n = 68 out of 387 (17.6%) Breast 19.2% Gastrointestinal 13.9% Genitourinary 17.0% Gynecologic 22.1% Lung 0% Hematologic 7.8%	The influence of NAFLD as an independent predictor of all-cause and cause-specific mortality among cancer survivors.	Subgroup analyses—overweight or obese (BMI > 25 kg/m^2^) cancer survivors with NAFLD were more likely to die than normal weight (BMI < 25 kg/m^2^) cancer survivors with NAFLD.	Among 387 cancer survivors, 17.6% had NAFLD. NAFLD was associated with an increased risk of all-cause mortality (HR = 2.52, 95% CI 1.47–4.34; *p* = 0.001) and cancer-specific mortality (HR = 3.21, 95% CI 1.46–7.07; *p* = 0.004). Younger cancer survivors (<60 years) with NAFLD were more likely to die than older cancer survivors with NAFLD (HR = 3.15, 95% CI 1.42–6.97; *p* = 0.005). Patients who had NAFLD had higher fasting insulin levels (117.0 vs. 62.2 pmol/L; *p* = 0.001), higher degrees of insulin resistance (5.9 vs. 2.8; *p* = 0.012), higher BMI (31.1 vs. 25.4 kg/m^2^; *p* < 0.001), wider WC (106.4 vs. 89.5 cm; *p* < 0.001) compared to those without NAFLD.
Nseir et al., 2017 Israel [37]	Case control study 4 years	n = 146 n (cases) = 73 Age (cases) = 54.8 ± 12 n (controls) = 73 Age (controls) = 57.5 ± 9.6	Breast Cancer	Abdominal CT	n = 33 out of 73, (45.2%)	Exploring correlation between NAFLD and BC.	BMI = 29.7	NAFLD was prevalent in 33 out of 73 women with BC and in 12 out of 73 controls (45.2% vs. 16.4%, respectively, *p* = 0.002). Multivariate analysis showed NAFLD (odds ratio 2.82, 95% confidence interval 1.2–5.5, *p* = 0.016) to be associated with BC.
Lee et al., 2017 Korea [33]	Cross sectional study	n = 104 Age (control): 49.29 ± 9.11 Age (NAFLD): 57.16 ± 11.51 BMI (control): 22.83 ± 2.92 BMI (NAFLD): 26.72 ± 5.17 F: 100%	Breast cancer	Liver MRI using Achieva 3.0 TX MRI scanner Fat signal percentage cut off of 5% to denote steatosis	n = 19 out of 104 (18.3%)	Evaluate the prevalence of NAFLD in breast cancer patients and compared it with the reported prevalence of NAFLD in general population.	Multivariate analysis—factors associated with NAFLD were high BMI OR = 1.403; 95% CI: 1.111–1.771; *p* = 0.05 30 obese patients with BMI > 25 kg/m^2^, 10 (33.3%) had NAFLD, whereas 74 patients with BMI < 25 kg/m^2^, only 9 (12.2%) had NAFLD.	19 out of 104 breast cancer patients were diagnosed with NAFLD (18.3%) In multivariate analysis, factors associated with NAFLD were high BMI (OR = 1.403; 95% CI: 1.111–1.771; *p* = 0.005), type 2 diabetes (OR = 11.872, 95% CI: 1.065–132.373; *p* = 0.004), and elevated triglycerides (OR = 50.267; 95% CI: 4.409–573.03; *p* = 0.002).
Bilici et al., 2007 Turkey [38]	Case-control study 1 year	n = 165 Group 1: 40 newly diagnosed, previously untreated breast cancer Group 2: 45 cases of treated with systemic therapy. Group 3: 40 cases of ovarian cancer. Group 4: 40 healthy women Mean age: Group 1 = 47.5 ± 11.9 Group 2 = 48.5 ± 11.6 Group 3 = 49.6 ± 11.8 Group 4 = 43.4 ± 6.0	Breast cancer, Ovarian cancer Treatment: Chemotherapy, Tamoxifen	Sonography	Group 1: n = 25 out of 40 (63%) Group 2: n = 33 out of 45 (72%)	Evaluated the influence of primary disease and treatment on steatosis in breast cancer. In addition, rate of steatosis in breast cancer cases was compared with a different solid cancer group and healthy population.	No BMI outcomes reported	Detected steatosis in 63%, 72%, 77%, and 48% of patients in groups 1, 2, 3, and 4, respectively. Steatosis was more frequent in breast cancer patients (group 1 and 2). Correlation was found between tamoxifen use and chemotherapy on development of non-alcoholic fatty liver disease. Detection of hepatic steatosis was seen in 83.3%, 84.3%, and 77.7% of cases with DM, obesity, and hypertriglyceridemia, respectively.
Aktas et al., 2014 Turkey [39]	Retrospective case control study 5 years	n (CRC) = 105 M = 65, F = 40 Mean age = 60.17 ± 12 years n (control) = 94 M = 48, F = 46 Mean age = 59.27 ± 16.4 years	Colorectal cancer	Abdominopelvic computed tomography images Serum transaminase	n = 21 out of 105 (20%) in CRC group	Retrospectively determine the relationship between non-alcoholic fatty liver disease and colorectal cancer by evaluating patients who underwent scanning or diagnosis colonoscopy	No BMI outcomes reported	The liver density measurement on contrast abdominopelvic computed tomography of CRC patients was low, indicating NAFLD. Although the ALT values were higher in the patient group, there was no statistical significance. In 21 patients (20%) of the CRC patient group, non-fatty areas were determined in the anterior of the portal vein (n = 15, 71.4%) and adjacent to the gallbladder (n = 6, 28.6%)
Molla et al., 2017 Canada [23]	Retrospective cohort study 27.5 months	n = 60 Age: 68.5 years BMI: 26.5 kg/m^2^ F: 40%, M: 60%	Colorectal cancer Treatment: liver resection, hepatectomy for colorectal cancer. 32 patients underwent right hepatectomy (53.33%), 9 underwent left hepatectomy (15%), 10 underwent left lateral hepatectomy (16.66%), and only 3 patients underwent right tri-segmentectomy. The rest either underwent wedge resection, single segmentectomy, or bi-segmentectomy.	The histologic features of NAFLD were scored using the NAFLD activity score, and the degree of fibrosis was determined. Preoperative and postoperative (at 12 weeks or more after surgery) CT imaging was retrieved for each patient.	n = 23 out of 60 (38.33%)	Examine the correlations between the degree of NAFLD, liver regeneration, and tumour recurrence after hepatectomy for colorectal cancer.	No BMI outcomes reported	The hepatic recurrence rate was 38.33%. Multivariate analysis revealed significant correlations of hepatic disease-free survival with hepatocyte ballooning (*p* = 0.0009), lesion diameter (*p* = 0.014), and synchronous disease (*p* = 0.006). Univariate and multivariate analyses did not reveal any correlation with degree of steatosis or recurrence rate.
Wu et al., 2019 United States [24]	Retrospective cohort study 10 years 6.6 years	n = 3262 Age range: 18–80 years	Colorectal cancer, stage I–III Treatment: surgical resection	Non-enhanced computed tomography scan	n = 83 out of 3262 (2.5%)	To examine the association between pre-existing NAFLD and CRC-specific mortality in stage I–III CRC patients utilizing data from the C- SCANS (Colorectal Cancer-Sarcopenia and Near-term Survival) project.	Associations were independent of BMI and were similar when the study restricted analysis to obese patients. Results for total mortality were similar when restricted analysis to CRC patients who were obese (BMI ≥ 30 kg/m^2^, 38 NAFLD cases, and 14 deaths; HR 1.79, 95% CI 1.03–3.11). Study did not examine associations separately for normal weight and overweight patients because of small number of deaths among CRC patients with NAFLD.	Cases diagnosed with NAFLD before and within 1 month after CRC diagnosis (pre-existing NAFLD; n = 83) had a HR of 1.64 (95% CI 1.06–2.54) for overall and a HR of 1.85 (95% CI 1.03–3.30) for CRC-specific mortality compared to those without NAFLD. According to the Kaplan–Meier survival function, patients with NAFLD had a shorter survival time than those without NAFLD. Findings did not differ significantly by sex, stage, tumor location, and smoking status, and were also similar when restricted to obese patients only.
Hoffmann et al., 2015 Germany [34]	Cross sectional study 5 years	n = 19 Age: without steatosis: 23.7 years; NAFLD: 25.2 years F: 53%, M: 47%,	Childhood-onset craniopharyngioma Treatment: Methylphenidate, modafinil to treat secondary narcolepsy and sever daytime sleepiness	Analyses of liver density were performed by non-contrasted CT and blood serum parameters	n = 10 out of 19 (52.6%)	To detect the risk for NAFLD in childhood-onset craniopharyngioma	No significant differences were detected in BMI. Signs of steatosis hepatis were not associated with BMI in study cohort.	NAFLD occurs in about 50% of childhood-onset craniopharyngioma patients with hypothalamic involvement and is associated with elevated liver enzymes. 10 out of 19 patients were identified with steatosis hepatis—three of them with severe steatosis hepatis (mean HU < 20) and seven with a moderate steatosis (mean HU 20–45). A significant association was found between steatosis hepatis and elevated liver enzymes; AST *p* = 0.041; Gamma-GT *p* = 0.016; GLDH *p* = 0.006)
Kouzu et al., 2020 Japan [40]	Retrospective case control 8 years	n = 721 Mean age: 68.4 years Preoperative BMI: 21.7 ± 2.9 Postoperative BMI: 19.3 ± 2.9 F: 23%, M: 77%	Gastric cancer	Plane abdominal CT. The average CT attenuation values of five arbitrary regions of the liver parenchyma without vessels were measured.	n = 35 out of 721 (4.9%)	Identify the risk factors for NAFLD after gastrectomy for gastric cancer.	NAFLD occurred at a high rate in patients with a high BMI. Univariate analysis identified the following factor as being significantly associated with the incidence of NAFLD: preoperative BMI ≥ 25 kg/m^2^ The NAFLD group had significantly higher preoperative and postoperative (1 year after) BMI (*p* = 0.001) than the non-NAFLD group.	The incidence of postoperative NAFLD was 4.9% (35/721). Following factors were significantly associated with the incidence of NAFLD: age (*p* = 0.003), preoperative BMI ≥ 25 kg/m^2^ (*p* = 0.005), tumor depth of pT3 ≤ (*p* = 0.016), lymph node metastasis grade of pN2 ≤ (*p* = 0.017), cholecystectomy (*p* = 0.005), D2 lymphadenectomy (*p* = 0.014), adjuvant chemotherapy (*p* < 0.001), high preoperative cholinesterase serum level (*p*= 0.029), and low grade of preoperative FIB-4 index (*p* < 0.001). Independent risk factors for NAFLD 1 year after gastrectomy were chemotherapy (*p* < 0.001) and high preoperative cholinesterase serum level (*p* = 0.021).
Moeini et al., 2017 United States [41]	Retrospective Case control study 28.8 months	n = 714 Mean age: 53.1 years F: 100%	Endometrial cancer Treatment: Oophorectomy	Radiology reports to diagnose NAFLD. NAFLD was defined as abnormal liver function testing in addition to radiographic evidence of increased hepatic echogenicity on ultrasonography or attenuation of the liver on CT.	n = 181 out of 714 (25.4%)	The association between NAFLD and venous thromboembolism examined in patients with endometrial cancer.	BMI reported but no significant outcomes	NAFLD was seen in 181 (25.4%) cases. There was 1 (0.1%) case of cirrhosis related to NAFLD, and no NASH case was reported in this study cohort.
Ariizumi et al., 2014 Japan [25]	Retrospective Study 21 years	n (CoCC) = 29 n (ICC) = 130 Age = 65 years median	Cholangiocellular carcinoma (CoCC) Intrahepatic cholangiocarcinoma (ICC) Hepatic resection	CT scans or multidetector helical CT Ultrasonography	n = 2 out of 29 (6.9%)	Comparison of surgical outcomes was compared between patients with CoCC and ICC.	No BMI outcomes reported	The number of patients with chronic liver disease was significantly higher in the CoCC group than in the ICC group.
Chang et al., 2018 Taiwan [26]	Retrospective cohort study 1 year	n = 266 Age = 52.9 ± 8.1 BMI = 24.1 ± 4.1	Breast cancer Tamoxifen	Ultrasound examination	n = 39 out of 266 (14.7%)	Assessed the potential risk and protective factors for tamoxifen-related NAFLD among BC patients.	BMI of > 22 kg/m^2^ is a risk factor for tamoxifen-related fatty liver	From 266 patients: 11 (4.1%) presented with alleviation of fatty liver 93 (35.0%) with normal and no change 39 (14.7%) with fatty liver and no change 65 (24.4%) with normal changing to fatty liver
Izadpanhai et al., 2020 Iran [35]	Cross sectional study	n = 152 patients n (BC) = 85 n (Gastrointestinal cancer) = 67 Median age = 45–54 years Body Mass Index = 23.17 ± 4.52	Breast cancer Gastrointestinal cancer Chemotherapy	Sonography for fatty liver	n (BC) = 40 out of 85 (47.1%) n = (GIC) = 41 out of 67 (61.2%)	Determine the prevalence of fatty liver in breast and gastrointestinal cancer patients during and after chemotherapy treatment.	No significant relationship between chemotherapy-induced fatty liver and BMI (*p* = 0.17).	The frequency of fatty liver after chemotherapy was significantly higher in females than in males (52.4% and 34.7%, respectively, *p* = 0.04). No significant relationship between chemotherapy-induced fatty liver and age (*p* = 0.9), and the presence of metabolic syndrome (*p* = 0.4). The results indicate that chemotherapy was associated with a significantly increased risk of fatty liver, which was more in women than in men. The highest frequency of fatty liver was observed in patients treated with paclitaxel, FOLFOX, and ECF with 53.5%, 42.9%, and 29.2%, respectively (*p* = 0.09).
Lee et al., 2019 Korea [32]	Retrospective Cohort Study 8.4 years	n (breast cancer) = 253 Median age = 69 years BMI = 22.9 ± 2.4 n(controls) = 220 Median age = 69 years BMI = 24.3 ± 3.5	Breast Cancer Aromatase inhibitors	Hepatic steatosis index (HIS) The cutoff value of HIS > 36 was used to detect NAFLD with specificity of 92.4%	n = 175 out 440 (39.8%)	Evaluate the role of aromatase inhibitors on the development of NAFLD and liver fibrosis in post- menopausal patients with early breast cancer	No BMI outcomes reported	Inhibition of estrogen synthesis in postmenopausal women undergoing treatment (aromatase inhibitors) could increase the risk of NAFLD. HIS was significantly higher in the aromatase inhibitor-treated group (33.15 ±4.35 vs. 38.08 ± 8.03; *p* = 0.001), and the proportion of patients with HIS > 36 who were considered to have high probability of NAFLD was significantly larger in the aromatase inhibitor-treated patients (25.9% vs. 53.6%; *p* = 0.001).
Pan et al., 2016 Taiwan [31]	Retrospective cohort study 26.7 months	n = 406 Tamoxifen group = 266 Control group = 140 mean age 53.2 ± 8.2 years BMI = 24.1 ± 3.9	Breast cancer Tamoxifen treatment	Abdominal ultrasound	**Control** **n (Initial)** Normal = 87 (62.1%) Mild = 39 (27.9%) Moderate = 13 (9.3%) Severe = 1 (0.7%) **n (Follow-up)** Normal = 92(65.7%) Mild = 32(22.9%) Moderate = 16(11.4%) Severe = 0 **Tamoxifen** **n (Initial)** Normal = 158 (60.1%) Mild = 83 (31.6%) Moderate = 21 (8.0%) Severe = 1 (0.4%) **n (Follow-up)** Normal = 101 (38.0%) Mild = 68 (25.6%) Moderate = 76 (28.6%) Severe = 21 (7/9%)	Examine the effects of tamoxifen under pre-existing fatty liver conditions and evaluate the prevalence of tamoxifen-related impaired liver function.	No BMI outcomes reported	The tamoxifen group had a higher risk of newly developed fatty liver HR = 3.69; 95% confidence interval CI = 1.678.13), lower rate of improved fatty liver (HR = 0.33; 95% CI 0.15–0.75), and higher rate of worsened fatty liver (HR = 2.11; 95% CI 1.02–4.35).
Nemoto et al., 2001 Japan [43]	Case control study 5 years	n = 56	Breast Cancer Tamoxifen—oral tamoxifen (40 mg/day for 2 to 3 years) as adjuvant endocrine therapy with systemic chemotherapy	CT of spleen and liver	n = 19 out of 56 (33.9%)	Representative clinical features of tamoxifen-induced NASH	No significant different in inverse values of body weight between patients with hepatic steatosis and patients without hepatic steatosis.	19 out 56 patients developed hepatic steatosis within 2 years.
Golabi et al., 2017 United States [36]	Cross sectional study 4 months	n = 11,187 Age of diagnosis: 72 years F: 31%, M: 69%	HCC Treatment: liver transplantation, surgical resection, Trans arterial chemoembolization	4 categories of chronic liver diseases were identified using the ICD-9 codes: (1) hepatic C virus, (2) Hepatitis B virus, (3) alcoholic liver disease with codes and (4) non-viral and nonalcoholic cryptogenic liver disease.	Non-viral and non-alcoholic cryptogenic liver disease =1277 out of 11,187 (18.6%) Decompensated hepatic cirrhosis = 3768 out of 11,187 (33.7%)	Assess mortality within 2 years postdiagnosis among participants with HCC according to treatment modalities.	No BMI outcomes reported	34% HCC patients had decompensated cirrhosis and 9% had non-viral and nonalcoholic/cryptogenic liver disease. 17% of HCC patients treated with surgical resection had non-viral and nonalcoholic/cryptogenic liver disease. Presence of decompensated cirrhosis (HR: 1.84, 95% CI = 1.73–1.96) increased within 2 years mortality. HCC patients with NAFLD (1.11 times) were more likely to die within 2 years of diagnosis.
Mehta et al., 2013 USA [30]	Retrospective cohort study 8 years	n = 155 M = 122 F = 33 Median age = 60 years	Infiltrative hepatocellular carcinoma (iHCC) trans arterial chemoembolization	Contrast-enhanced computed tomography or magnetic resonance imaging	N = 15 out of 155 (9.7%)	Aim of the present study was to assess the outcomes, effect of treatment, and factors predicting prognosis in a large cohort of patients with iHCC.	No BMI outcomes reported	Nonalcoholic fatty liver disease (9.7%) Most of the patients had tumours of Barcelona Clinic Liver Cancer Stage C (70%) or D (22%). On multivariate analysis, predictors of 6-month mortality were Child–Pugh class B or C cirrhosis; lack of tumour-directed therapy with chemoembolization. The percentages of patients surviving 6 and 12 months were 17% and 2% for those who received no therapy (n = 109), 73% and 36% for those who received sorafenib (n = 11), and 45% and 17% for those who received trans arterial chemoembolization (n = 18) (all *p* values < 0.01).
Prieto et al., 2016 Philippines [29]	Retrospective cohort study 7 years	n = 346 Mean age: 61.47 + 13.08 F: 18.8%, M: 81.8%	Hepatocellular carcinoma Treatment: 44 patients (12.94%) underwent surgical treatment. 99 patients (29.12%) had TACE and/or RFA. 26 patients (7.65%) had systemic/oral chemotherapy. 171 (50.29%) patients had supportive care	Ultrasound, Dynamic CT scan, MRI using liver specific contrast, elevated AFP, and biopsy	n = 27 out of 346 (7.8%)	To investigate prognostic features, treatment outcomes and survival of hepatocellular carcinoma patients at the National Kidney and Transplant Institute.	No BMI outcomes reported	Median survival was 13.17 months (range, < one month—92 months). Those who had locoregional therapy had the longest median survival (30.33 months), followed by systemic chemotherapy (26.67 months) then surgery (13.17 months). Median survival time between those with and without liver cirrhosis was significantly different (9.43 months vs. 38.47 months, *p* < 0.001).
Bruno et al., 2005 Italy [44]	Prospective, randomized, double blind, placebo-controlled trial 8.7 years	n = 64 Median age = 51 years Median BMI = 27.0	Endometrial cancer Hysterectomies Tamoxifen	Ultrasonography	n = 52 suspected of having developed NAFLD (81.25%)	Assess the risk of development of non-alcoholic fatty liver disease, including non-alcoholic steatohepatitis, in relation to tamoxifen in women	Women with high alanine aminotransferase at baseline were heavier, had a higher BMI, and more often had diabetes than women with normal concentrations at baseline and during follow-up (*p* < 0.0001).	Developed non-alcoholic fatty liver disease = 52 (34 tamoxifen, 18 placebo)—hazard ratio = 2.0 (95% confidence interval 1.1 to 3.5; *p* = 0.04). Factors associated with the development NAFLD include overweight (2.4, 1.2 to 4.8), obesity (3.6, 1.7 to 7.6), hypercholesterolaemia (3.4, 1.4 to 7.8), and arterial hypertension (2.0, 1.0 to 3.8). Twenty women had liver biopsies: 15 were diagnosed as having mild to moderate steatohepatitis (12 tamoxifen, 3 placebo), and five had fatty liver alone (1 tamoxifen, 4 placebo).
Matuso et al., 2016 Japan [42]	Retrospective cohort study 1, 2 and 5 years follow-up	n (total) = 875 Oophorectomy cases = 712 No oophorectomy cases = 163 Mean age = 50.8 years	Endometrial cancer Surgical treatment	Ultrasonography	n = 232 out of 875 cases (26.5%)	Examine factors associated with development of NAFLD among women with endometrial cancer who underwent surgical staging	Women who developed NAFLD were obese (*p* = 0.029).	NAFLD was diagnosed in 232 cases (26.5%) at the time of endometrial tumour diagnosis or during follow-up after surgical operation. Prevalence of NAFLD in 875 women with endometrial tumour was 14.1%, 20.5%, and 38.4% at 1, 2, and 5 years after surgical operation, respectively. Oophorectomy in women with endometrial cancer significantly increases the risk of NAFLD. NAFLD cases were diagnosed after surgical operation (n = 168; 72.4%) and were commonly diagnosed by ultrasonography (76.2%).
Gild et al., 2018 Germany [28]	Cohort study 6.1 years	n = 82,938 No Androgen-deprivation therapy n = 51,821 Age = 72.2 (68.8–76.5) Androgen-deprivation therapy = 31,117 Age = 75.3 (71.1–80.0)	Prostate cancer Androgen-deprivation therapy	CT scan	n (No Androgen-deprivation therapy) = 259 out of 51,821 (0.5%) n (Androgen-deprivation therapy) = 265 out of 31,117 (0.9%)	Association between androgen-deprivation therapy and prostate cancer. The primary study outcome was the diagnosis of nonalcoholic chronic liver disease, including NAFLD and NASH.	No BMI outcomes reported	Of the men who underwent androgen-deprivation therapy, they were most likely to be diagnosed with nonalcoholic fatty liver disease (HR 1.54, 95% CI 1.40–1.68), liver cirrhosis (HR 1.35, 95% CI 1.12–1.60), liver necrosis (HR 1.41, 95% CI 1.15–1.72) and any liver disease (HR 1.47, 95% CI 1.35–1.60).
Montomoli et al., 2013 Denmark [27]	Cohort study 14 years 30 days	n = 39,840 Non-cirrhotic liver disease = 369 F: 48.9%, M: 51.1% Liver cirrhosis = 158 F: 49.1%, M: 50.1%	Colorectal cancer Treatment: Colorectal surgery—radical resection, laparoscopic and open surgery.	Danish National Registry of Patients to identify patients with a diagnosis of liver disease.	n = 34 out of 369 (9.2%)	Examined 30-day mortality after CRC surgery in patients with liver disease compared to those without liver disease.	No BMI outcomes reported	Thirty-day mortality was 13.3% in patients with non-cirrhotic liver disease and 24.1% among patients with liver cirrhosis, compared to 8.7% in patients without liver disease. Patients with liver cirrhosis, mortality was 24.1%, Adjusted RR = 2.59, 95% CI: 1.86–3.61 CRC patients with liver disease, especially those with liver cirrhosis, were more likely to have comorbid conditions, including non-hepatic alcohol-related disease, than patients without liver disease.

NR, not reported; NA, not applicable; BMI, body mass index; WC, waist circumference; F, female; M, male; HSCT, haematopoietic stem cell transplantation; CT, computed tomography; AST, aspartate transaminase; GAMMA-GT, gamma-glutamyl transferase; GLDH, glutamate dehydrogenase; CRC, colorectal carcinoma; CoCC, cholangiocellular carcinoma; ICC, intrahepatic cholangiocarcinoma; DM, diabetes mellitus; T2DM, type 2 diabetes mellitus; EILD, eribulin-induced liver dysfunction.

**Table 3:** Study outcomes related to treatment modalities, survival and mortality and co-morbidities and risk of NAFLD ^1^.

Author, Year	Cancer Treatment: Chemotherapy	Cancer Treatment: Hormone Therapy	Cancer Treatment: Surgery	Survival and Mortality	Metabolic Co-Morbidities
Brown et al., 2017 [22]	−	−	−	+	+
Nseir et al., 2017 [37]	−	−	−	−	
Lee et al., 2017 [33]	−	−	−	−	+
Bilici et al., 2007 [38]	+	−	−	−	+
Aktas et al., 2014 [39]	−	−	−	−	−
Molla et al., 2017 [23]	−	−	−	+	−
Wu et al., 2019 [24]	−	−	−	−	+
Hoffmann et al., 2015 [34]	−	−	−	−	+
Kouzu et al., 2020 [40]	−	−	−	−	+
Moeini et al., 2017 [41]	−	−	+	−	−
Ariizumi et al., 2014 [25]	−	−	−	−	−
Chang et al., 2018 [26]	−	+	−	−	−
Izadpanhai et al., 2020 [35]	+	−	−	−	−
Lee et al., 2019 [32]	−	+	−	+	−
Pan et al., 2015 [31]	+	+	−	−	−
Nemoto et al., 2001 [43]	−	+	−	−	−
Golabi et al., 2017 [36]	+	−	+	+	−
Mehta et al., 2013 [30]	−	−	−	−	−
Prieto et al., 2016 [29]	−	−	−	−	−
Bruno et al., 2005 [44]	−	+	−	−	−
Matuso et al., 2016 [42]	−	−	+	−	+
Gild et al., 2018 [28]	−	+	−	−	−
Montomoli et al., 2013 [27]	−	−	−	+	+

^1^ The positive sign (+) indicates study included outcome and negative sign (−) indicates study did not include outcome. Prevalence of NAFLD in individuals diagnosed with cancer.

**Table 4:** Critical appraisal of the 23 studies with the use of Quality Criteria Checklist ^1^.

Aktas et al., 2014 [39]	Ariizumi et al., 2014 [25]	Bilici et al., 2007 [38]	Brown et al., 2017 [22]	Chang et al., 2018 [26]	Gild et al., 2018 [28]	Golabi et al., 2017 [36]	Hoffmann et al., 2015 [34]	Izadpanahi et al., 2020 [35]	Kouzu et al., 2020 [40]	Lee et al., 2019 [32]	Lee et al., 2017 [33]	Moeini et al., 2017 [41]	Matuso et al., 2016 [42]	Molla et al., 2017 [23]	Mehta et al., 2013 [30]		Montomoli et al., 2013 [27]	Nemoto et al., 2002 [43]	Nseir et al., 2017 [37]	Pan et al., 2016 [31]	Prieto et al., 2016 [29]	Wu et al., 2019 [24]
																Relevance Questions						
NA	NA	NA	NA	NA	NA	NA	NA	NA	NA	NA	NA	NA	NA	NA	NA	1. Would implementing the studied intervention or procedure (if found successful) result in improved outcomes for the patients/clients/population group?	NA	NA	NA	NA	NA	NA
Y	Y	Y	Y	Y	Y	Y	Y	Y	Y	Y	Y	Y	Y	Y	Y	2. Did the authors study an outcome (dependent variable) or topic that the patients/clients/population group would care about?	Y	Y	Y	Y	Y	Y
NA	NA	NA	NA	NA	NA	NA	NA	NA	NA	NA	NA	NA	NA	NA	NA	3. Is the focus of the intervention or procedure (independent variable) or topic of study a common issue of concern to dietetics practice?	NA	NA	NA	NA	NA	NA
NA	NA	NA	NA	NA	NA	NA	NA	NA	NA	NA	NA	NA	NA	NA	NA	4. Is the intervention or procedure feasible?	NA	Y	Y	Y	NA	NA
																**Validity Questions**						
Y	Y	Y	Y	Y	Y	Y	Y	Y	Y	Y	Y	Y	Y	Y	Y	1. Was the research question clearly stated?	Y	Y	Y	Y	Y	Y
Y	Y	Y	Y	Y	Y	Y	Y	Y	Y	Y	Y	Y	Y	Y	Y	2. Was the selection of study subjects/patients free from bias?	Y	U	Y	Y	Y	Y
Y	NA	NA	NA	Y	NA	NA	NA	NA	NA	Y	NA	NA	NA	NA	NA	3. Were study groups comparable or was an appropriate reference standard used?	NA	NA	NA	NA	NA	NA
NA	NA	NA	NA	NA	U	NA	U	NA	NA	Y	NA	U	Y	NA	Y	4. Were methods of handling losses from the original sample (withdrawals) described?	NA	NA	NA	NA	NA	U
NA	NA	NA	NA	NA	NA	NA	NA	NA	NA	NA	NA	NA	NA	NA	NA	5. Was blinding used to prevent introduction of bias?	NA	NA	NA	NA	U	NA
NA	NA	NA	NA	NA	NA	NA	NA	Y	NA	Y	NA	NA	Y	NA	Y	6. Was the intervention/treatment regimen/exposure factor, procedure, process or product of interest, and any comparison(s) described in detail? Were intervening factors described?	NA	Y	Y	Y	Y	Y
Y	Y	Y	Y	Y	Y	Y	Y	Y	Y	Y	Y	Y	Y	Y	Y	7. Were outcomes or condition or status of interest clearly defined and the measurements validand reliable?	Y	N	Y	Y	Y	Y
Y	Y	Y	Y	Y	Y	Y	Y	Y	Y	Y	Y	Y	Y	Y	Y	8. Was the statistical analysis appropriate for the study design and type of outcome indicators?	Y	N	Y	Y	Y	Y
Y	Y	Y	Y	Y	Y	Y	Y	Y	Y	Y	Y	Y	Y	Y	Y	9. Are conclusions supported by results with biases and limitations taken into consideration?	Y	Y	Y	Y	Y	Y
Y	Y	Y	Y	Y	Y	Y	Y	Y	Y	Y	Y	Y	Y	Y	Y	10. Is bias due to study’s funding or sponsorship unlikely?	Y	N	N	N	N	Y
**+**	**Ø**	**Ø**	**Ø**	**+**	**Ø**	**Ø**	**Ø**	**+**	**Ø**	**+**	**Ø**	**Ø**	**+**	**Ø**	**+**	**Overall Rating**	**Ø**	**Ø**	**+**	**+**	**+**	**+**

^1^ Abbreviations: NA = Not Applicable; U = Unclear. Positive (+) = most of the answers to the validity questions are “Yes” (including criteria 2, 3, 6, and 7 and at least one additional “Yes”). Neutral (Ø) = the answers to the validity criteria questions 2, 3, 6, and 7 do not indicate that study is exceptionally strong. Negative (−) = most (six or more) of the answers to the validity questions are “No”.

**Table 5:** Risk of bias of randomized controlled trials according to the Cochrane Risk of Bias Tool 2.0.

Author	Randomized Process	Deviations from Intended Interventions	Missing Outcome Data	Measurement of the Outcome	Selection of the Reported Result	Overall
Bruno et al., 2005 [44]	Some concerns	High risk	Low risk	Some concerns	Some concerns	Some concerns

## Supplementary Materials

The following supporting information can be downloaded at: https://www.mdpi.com/article/10.3390/curroncol30010005/s1, Table S1: PRISMA 2020 Checklist.

## Appendix A

### Search Strategy

The search strategy was using a keyword search of the following terms, including Medical Subject Headings:

1. ‘survivor’ OR ‘survivors’ OR ‘patient’ OR ‘rehabilitation’ AND
2. ‘cancer’ OR ‘cancer[MESH terms]’ OR ‘neoplasms[MESH terms]’) OR ‘breast cancer’ OR ‘colorectal cancer’ OR ‘prostate cancer’ OR ‘hepatocellular cancer’ OR ‘liver cancer’ OR ‘gastrointestinal cancer’ OR ‘gastric cancer’ OR ‘endometrial cancer’ OR ‘ovarian cancer’ OR ‘renal cancer’ OR ‘kidney cancer’ OR ‘hepatic cancer’ OR ‘genitourinary cancer’ OR ‘gynaecologic cancer’ OR ‘lung cancer’ OR ‘hematologic cancer’ OR ‘bladder cancer’ OR ‘rectal cancer’ OR ‘leukemia’ OR ‘lung cancer’ OR ‘pancreatic cancer’ OR ‘thyroid cancer’ OR ‘osteosarcoma’ OR ‘nasopharyngeal cancer’ OR ‘cervical cancer’ OR ‘nasopharyngeal cancer’ OR ‘skin cancer’ AND
3. ‘nafld’ OR ‘nash’ OR ‘fatty liver’ OR ‘liver disease’ OR ‘non-alcoholic fatty liver’ OR ‘nonalcoholic fatty liver’ OR ‘non-alcoholic steatosis’ OR ‘nonalcoholic steatosis’ OR ‘steatosis’ OR ‘mafld’ OR ‘metabolic associated fatty liver’ OR ‘metabolic-associated fatty liver’.
